# Prognostic Value of CD44 and Its Isoforms in Advanced Cancer: A Systematic Meta-Analysis With Trial Sequential Analysis

**DOI:** 10.3389/fonc.2019.00039

**Published:** 2019-02-06

**Authors:** Susu Han, Tao Huang, Wen Li, Xiyu Wang, Xing Wu, Shanshan Liu, Wei Yang, Qi Shi, Hongjia Li, Fenggang Hou

**Affiliations:** ^1^Shanghai Municipal Hospital of Traditional Chinese Medicine, Shanghai University of Traditional Chinese Medicine, Shanghai, China; ^2^The Affiliated Hospital of Ningbo University, Ningbo, China

**Keywords:** advanced cancer, CD44v9, prognosis, CD44, therapy

## Abstract

**Objective:** Cancer stem cell marker CD44 and its variant isoforms (CD44v) may be correlated with tumor growth, metastasis, and chemo-radiotherapy resistance. However, the prognostic power of CD44 and CD44v in advanced cancer remains controversial. Therefore, the purpose of our study was to generalize the prognostic significance of these cancer stem cell markers in advanced cancer patients.

**Methods:** Hazard ratios (HRs) with 95% confidence intervals (95% CIs) were calculated from multivariable analysis to assess the associations among CD44, CD44v6, and CD44v9 positivity and overall survival (OS), disease-free survival (DFS), progression-free survival (PFS), cancer-specific survival (CSS), and recurrence-free survival (RFS). Trial sequential analysis (TSA) was also conducted.

**Results:** We included 15 articles that reported on 1,201 patients with advanced cancer (CD44: nine studies with 796 cases, CD44v6: three studies with 143 cases, and CD44v9: three studies with 262 cases). CD44 expression was slightly linked to worse OS (HR = 2.03, *P* = 0.027), but there was no correlation between CD44 expression and DFS, RFS, or PFS. Stratified analysis showed that CD44 expression was not correlated with OS at ≥5 years or OS in patients receiving adjuvant therapy. CD44v6 expression was not associated with OS. CD44v9 expression was closely associated with poor 5-years CSS in patients treated with chemo/radiotherapy (HR = 3.62, *P* < 0.001). However, TSA suggested that additional trials were needed to confirm these conclusions.

**Conclusions:** CD44 or CD44v9 might be novel therapeutic targets for improving the treatment of advanced cancer patients. Additional prospective clinical trials are strongly needed across different cancer types.

## Introduction

Cancer remains a pressing worldwide health issue (1), although surgery, chemotherapy, radiotherapy therapy, and targeted molecular therapy have greatly changed clinical outcomes for cancer patients in recent years. Advanced cancer patients (advanced-stage or metastatic disease) are resistant to chemotherapy, radiotherapy and targeted therapies (due to secondary mutations). Thus, effective treatment strategies for advanced cancer patients are still limited and disappointing. The prognosis of patients with advanced cancer remains very poor such as a low 5-years survival rate (2–6). For all cancers combined, the 5-years survival rate is >60%. For different cancer types, it varies; for example, the 5-years survival rate is 14% for advanced colorectal cancer and 29% for advanced ovarian cancer (7). Therefore, novel molecular therapeutic targets to prolong survival in advanced cancer are warranted and could help physicians to stratify cancer patients.

Increasing evidence has suggested that cancer stem cells (CSCs) represent a small subset of cancer cells with the major capabilities of self-renewal and multidifferentiation and may be responsible for tumor relapse, metastasis, progression, a poor prognosis, and resistance to chemotherapy or radiation therapy (8, 9). Several CSC marker molecules, such as CD166, epithelial cell adhesion molecule (EpCAM) and CD44, have been identified and have become useful markers in human cancers (10–12). Among these CSCs, the CD44 family is one of the most commonly reported markers in cancer.

CD44 was originally described as a hyaluronan and lymphocyte-homing receptor (13). Exons 1–5 and 16–20 produce the standard form of CD44 and the remaining exons 6–15 encode variant exons 1–10 (v1–v10). The isoform with none of the 10 variable exons is denoted CD44 standard (CD44s) and the alternative mRNA splicing of variant isoforms of CD44 are named CD44v. CD44 has been reported to be involved in the regulation of cell growth, survival, differentiation, motility, tumor growth, proliferation, and metastasis (14–16). CD44s and CD44v have overlapping and distinct functions. CD44v contains additional binding motifs that can contribute to the interaction of CD44 with molecules in the microenvironment (17). The pattern of CD44 alternative splicing is differentially regulated during epithelial-mesenchymal transition (EMT). EMT is an important step in the metastatic process and the acquisition of stemness in cancer cells. CD44s is more highly expressed by EMT CSCs, and CD44v is more highly expressed by non-EMT CSCs (14, 18). CD44 or CD44v6 expression has been shown to be correlated with poor overall survival (OS) in gastric cancer (19), but CD44 or CD44v6 expression has been found to not be correlated with OS in ovarian cancer (20); these findings suggest that some prognostic information regarding CD44 is still conflicting.

The role of CD44 and its isoforms in advanced cancer patients remains unclear as to which markers may be of value in determining prognosis. Therefore, we present here the first systemic meta-analysis and TSA on the relationship of CD44 and its isoforms expression with clinical outcomes in patients with advanced cancer.

## Materials and Methods

### Search Strategies

Meta-analysis was performed in accordance with the Preferred Reporting Items for Systematic Review and Meta-analysis (PRISMA) statement (21). A systematic literature search was conducted in the PubMed, EMBASE, EBSCO, Web of Science, and Cochrane Library databases through April 2018 without language restrictions. The following key words and search terms were used as follows: “CD44,” “metastatic OR advanced OR metastasized OR recurrent,” “cancer OR tumor OR carcinoma OR neoplasm,” “survival OR outcome OR prognosis.” Moreover, the references listed in the eligible articles were also manually searched to avoid the omission of relevant papers.

### Selection Criteria

Eligible publications were included when the given eligibility criteria were satisfied: (1) studies reporting patients with advanced/metastatic cancer or stage III cancer or stage IV cancer; (2) studies investigating the prognostic value of the expression of CD44 and its isoforms using immunohistochemical (IHC) assays; **(**3) studies reporting multivariable survival analysis with hazard ratio (HR) with 95% confidence interval (CI) for overall survival (OS), disease-free survival (DFS), progression-free survival (PFS), relapse/recurrence-free survival (RFS), metastasis-free survival (MFS) or cancer-specific survival (CSS); (4) In the case of insufficient data, such as only *P*-value with HR or only the 95% CI, HR and 95% CI were calculated using the previously described method (22, 23), or the corresponding author with an available email was contacted to request the relevant information. Case reports, reviews, comments, letters, cell lines, animal studies, articles unrelated to our topic, studies with no available prognostic data and studies with advanced cancer patients analyzed using univariable survival analysis were excluded. We did not include overlapping sample data in multiple publications from the same research institution.

### Data Extraction and Study Assessment

The study assessment was conducted following Reporting Recommendations for Tumor Marker Prognostic Studies (REMARK) guidelines (24). The REMARK checklist reported 20 items for published tumor prognostic markers, consisting of Introduction (1 item), Materials and Methods (10 items), Results (7 items), and Discussion sections (2 items), with a maximum score of 40 (Table S1). An item had possible scores of 0, 1, and 2. An item was given a score of 2 if it described all aspects of an item, a score of 1 if it reported some aspects and an item was given a score of 0 when an item was lacking reporting of any aspect. Multivariable survival analysis adjusted for potential factors such as traditionally prognostic factors is more valuable compared to a study that reported a univariable survival analysis. Therefore, this meta-analysis only included prognostic information obtained using multivariable analysis. The following data items were extracted from eligible full-text papers: first author's surname, year of publication, number of patients, study source of patients, mean or median age, tumor type, detection method, therapy regime, study design, sample type, cut-off value, median or mean follow-up time, survival rate, adjusted factors, and clinical outcomes. Any inconsistency was discussed until a consensus was reached.

### Statistical Analysis

Pooled HR and 95% CI were calculated to estimate the prognostic effect of the expression of CD44 and its isoforms on patients with advanced cancer, including OS, DFS, PFS, CSS, RFS, or MFS using multivariable analysis. A HR >1 showed a worse survival, whereas an observed HR <1 showed a favorable survival. Heterogeneity was tested using Cochran's Q statistic (25), with *P* < 0.1 indicating substantial heterogeneity. The random-effects model (DerSimonian-Laird) was applied to estimate the HR (26, 27). Subgroup analyses were performed in ≥8 of the included studies and publication bias was measured by using the Egger's and Begg's funnel plots (28, 29).

Since the meta-analysis included only a small number of patients and the associated random errors may cause spurious results (30, 31), trial sequential analysis (TSA) was performed to control for random errors and to assess the required sample information (32). The relative risk reduction (RRR) of 20% was applied for the minimum intervention effect. Type I error (α) level of 5%, type II error (β) level of 20% (giving a statistical power of 80%) and the optimal a priori anticipated information size (APIS) method were used. Monitoring boundaries were applied to decide whether a trial could be terminated early. When the cumulative Z-curve passed through the trial sequential monitoring boundary or required information size (RIS) boundary, this suggested the evidence was conclusive and reliable. Otherwise, additional clinical studies are essential. Meta-analyses were performed by using Stata software, version 12.0 (Stata Corp., College Station, TX, USA) and R software, version 3.4.2 (The R Foundation for Statistical Computing; Vienna, Austria).

## Results

### Study Characteristics

**Figure 1:**
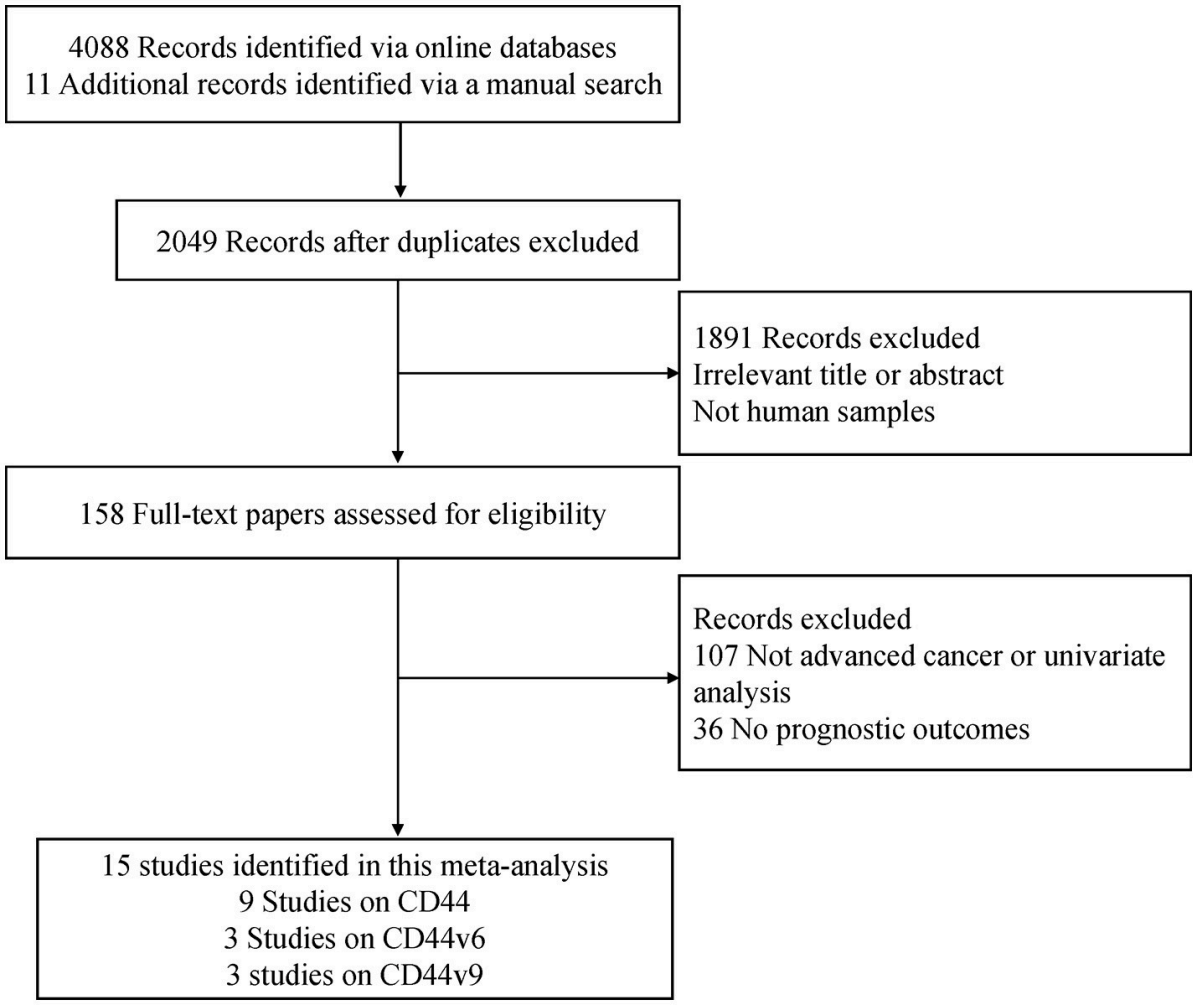
Flow diagram of the study selection.

A flow chart of the literature search strategies is shown in Figure 1. After carefully reviewing the titles, abstracts and full text, a total of 1,201 patients with advanced cancer from 15 full text articles published from 1999 to 2018 met the inclusion criteria and were included in the current meta-analysis (33–47). Six studies were conducted in Japan, four studies in the USA, two studies in Germany, one study in Greece, one study in Brazil, and one study in China. One study was a prospective trial and the remaining studies were retrospective in design. The mean REMARK scores were 19, ranging from 14 to 24.

The majority (14 articles) of the eligible 15 articles reported advanced cancer patients treated with surgery and/or adjuvant therapy. Nine studies involving 796 advanced cancer patients evaluated the association between CD44 expression and the prognosis (34–36, 40, 42–46) and only six studies evaluated 5-years survival. Three studies assessed the association of CD44v6 expression and 5-years prognosis (33, 38, 39), including 143 cases treated with surgery and/or adjuvant therapy. Three studies evaluated the correlation between CD44v9 expression and 5-years prognosis (37, 41, 47), including 262 cases treated with surgery and chemo/radiotherapy. The characteristics of the included studies using multivariable survival analysis are presented in Table 1 and Table S2.

**Table 1:** Main characteristics of studies included in the meta-analysis.

**References**	**Country**	**Age**	**Method**	**Cancer type**	**Study design**	**Cases**	**Therapy**	**Staining patterns**	**Cut off (positivity)**	**Survival rate**	**Outcomes**
**CD44**											
Rodriguez-Rodriguez et al. (34)	USA	NA	IHC	Metastatic ovarian cancer	Retrospective, single-center	87	NA	RandD Systems, MN; dilution 1:1000	Membrane/cytoplasm 0%	<5 years	DFS
Singh et al. (35)	USA	NA	IHC, blind	Metastatic or recurrent endometrial carcinoma	Prospective, Gynecologic Oncology Group	42	Tamoxifen combined with intermittent medroxyprogesterone acetate	Dako anti-CD44 clone DF1485	Cytoplasm median-high	NA	OS, PFS
Koukourakis et al. (36)	Greece	68	IHC, blind	Advanced squamous cell head-neck cancer	Retrospective, multicenter	74	Surgery and radio-chemotherapy	Ab6124, Abcam, Cambridge, UK; dilution 1:100	Membrane/cytoplasm 40%	5 years	RFS
Udagawa et al. (40)	Japan	66	IHC	Lung squamous cell carcinoma with lymph node metastasis	Retrospective, single-center	113	Surgery	Clone DF1485, Novocastra Laboratories; dilution 1:150	Median	5 years	RFS
Linge et al. (42)	Germany	NA	IHC, blind	Advanced head and neck squamous cell carcinoma	Retrospective, multicenter	195	Surgery and cisplatin-based radiochemotherapy	clone DF1485; Dako; dilution 1:500	1 score	5 years	OS, MFS
Ribeiro et al. (43)	Brazil	NA	IHC	Metastatic colon cancer	Retrospective, single-center	58	Chemotherapy	clone DF1485, DakoCytomation	Membrane and/or cytoplasm 3–6 scores	NA	OS, PFS
Baschnagel et al. (44)	USA	61	IHC, blind	Advanced head and neck squamous cell carcinoma	Retrospective, NA	105	Chemoradiotherapy	Clone EPR1013Y, Abcam, Cambridge, MA, USA, dilution 1:50	50% of tumor cells	5 years	DFS
Sun et al. (45)	China	NA	IHC	Breast cancer with axillary lymph node metastasis	Retrospective, single-center	59	Surgery	Peking Zhongshan Biotechnology Limited Company, China	>3 scores	>5 years	OS
Boxberg et al. (46)	Germany	NA	IHC	Advanced oral squamous cell carcinoma	Retrospective, single-center	63	Surgery and adjuvant radiotherapy	Cell Signaling, clone 156-3C11; dilution 1:100	Membrane 9–12 scores	5 years	CSS, OS, DFS
**CD44v9**											
Aso et al. (37)	Japan	61	IHC	Advanced head and neck squamous cell carcinoma	Retrospective, National Kyushu Cancer Center	102	Surgery and chemoradiotherapy	RV3; dilution 1:12,500	2–5 scores	5 years	CSS
Hagiwara et al. (41)	Japan	NA	IHC, blind	Advanced upper tract urothelial cancer	Retrospective, single-center	83	Surgery and adjuvant cisplatin-based chemotherapy	CosmoBio, Tokyo, Japan; dilution 1:5,000	Cell membrane 5%	5 years	CSS, RFS
Hagiwara et al. (47)	Japan	68	IHC, blind	Metastatic and/or recurrent urothelial cancer	Retrospective, single-center	77	Surgery and cisplatin-based chemotherapy	Cosmo Bio, Tokyo, Japan; dilution 1:5,000	Cell membrane 5%	5 years	CSS
**CD44v6**											
Fukuse et al. (33)	Japan	64	IHC, blind	Metastatic non-small cell lung carcinoma	Retrospective, NA	34	Surgery and adjuvant therapies	VFF-18; Bender Co; dilution 1:100	Cell membrane 20%	5 years	OS
Marzese et al. (38)	USA	62	IHC	Metastatic melanoma	Retrospective, single-center	50	Surgery	H-CAM; Santa Cruz Biotechnology, Santa Cruz, CA	27	5 years	PFS
Tjhay et al. (39)	Japan	57	IHC	Advanced epithelial ovarian cancer	Retrospective, single-center	59	Surgery and chemotherapy	2F10; RandD Systems, Minneapolis, MN, USA	10%	5 years	OS

*IHC, immunohistochemistry; NA, not applicable; OS, overall survival; DFS, disease-free survival; PFS, progression-free survival; CSS, cancer-specific survival; RFS, recurrence-free survival (RFS); MFS, metastasis-free survival*.

### Association Between CD44 Expression and the Prognosis

**Figure 2:**
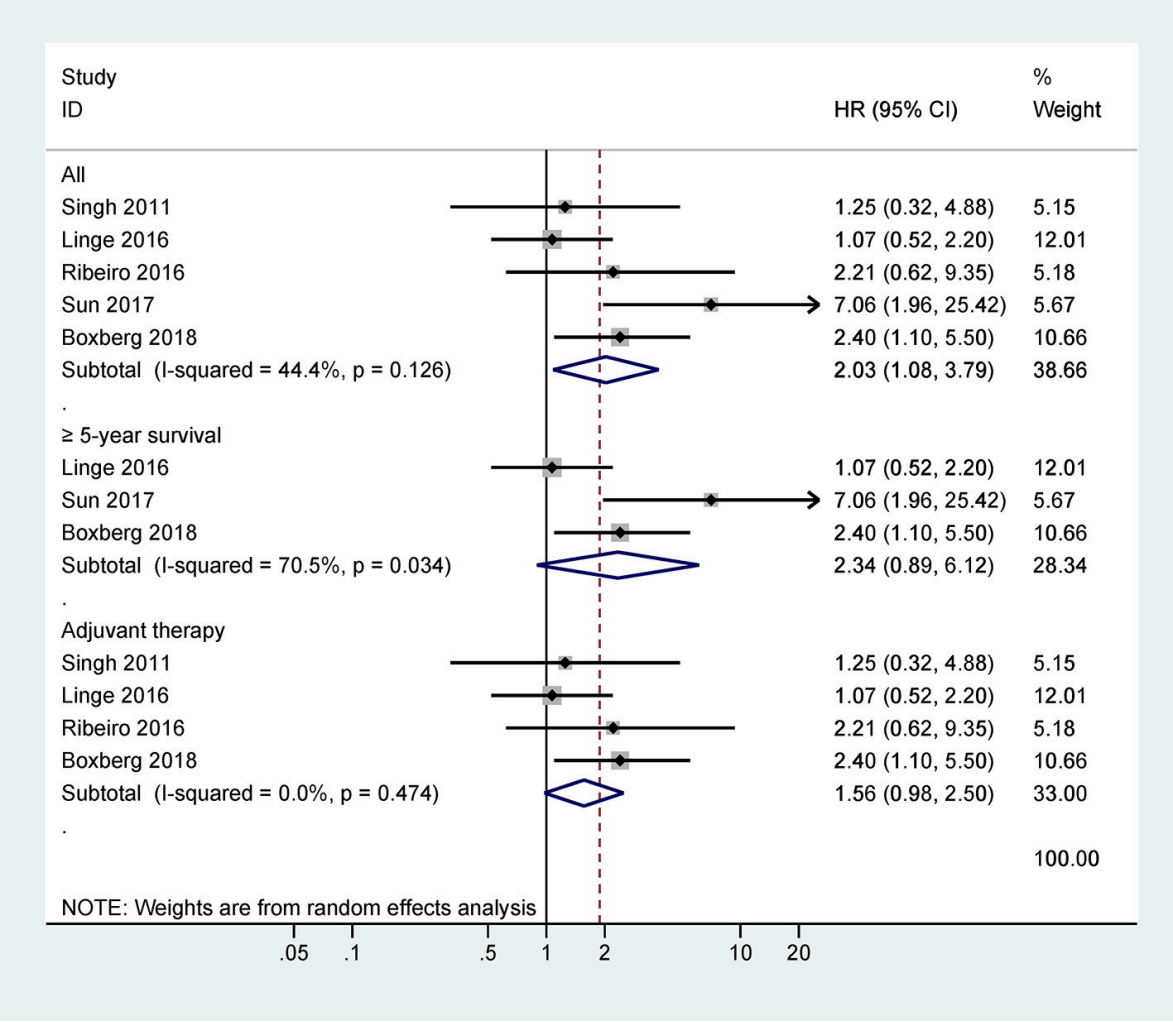
Forest plot for the association between CD44 expression and overall survival (OS).

Five studies with 417 cases were included in the final analysis of CD44 expression and OS and the pooled data showed that CD44 expression was associated with worse OS (HR = 2.03, 95% CI = 1.08–3.79, *P* = 0.027) (Figure 2), with no obvious evidence of heterogeneity (*P* = 0.126).

Further analysis from three studies with 317 cases indicated that CD44 expression was not associated with OS at ≥ 5 years (HR = 2.34, 95% CI = 0.89–6.12, *P* = 0.084) (Figure 2). Data from four studies with 358 cases receiving adjuvant therapy showed that no significantly statistical association was observed between CD44 expression and OS (HR = 1.56, 95% CI = 0.98–2.50, *P* = 0.062) (Figure 2).

**Figure 3:**
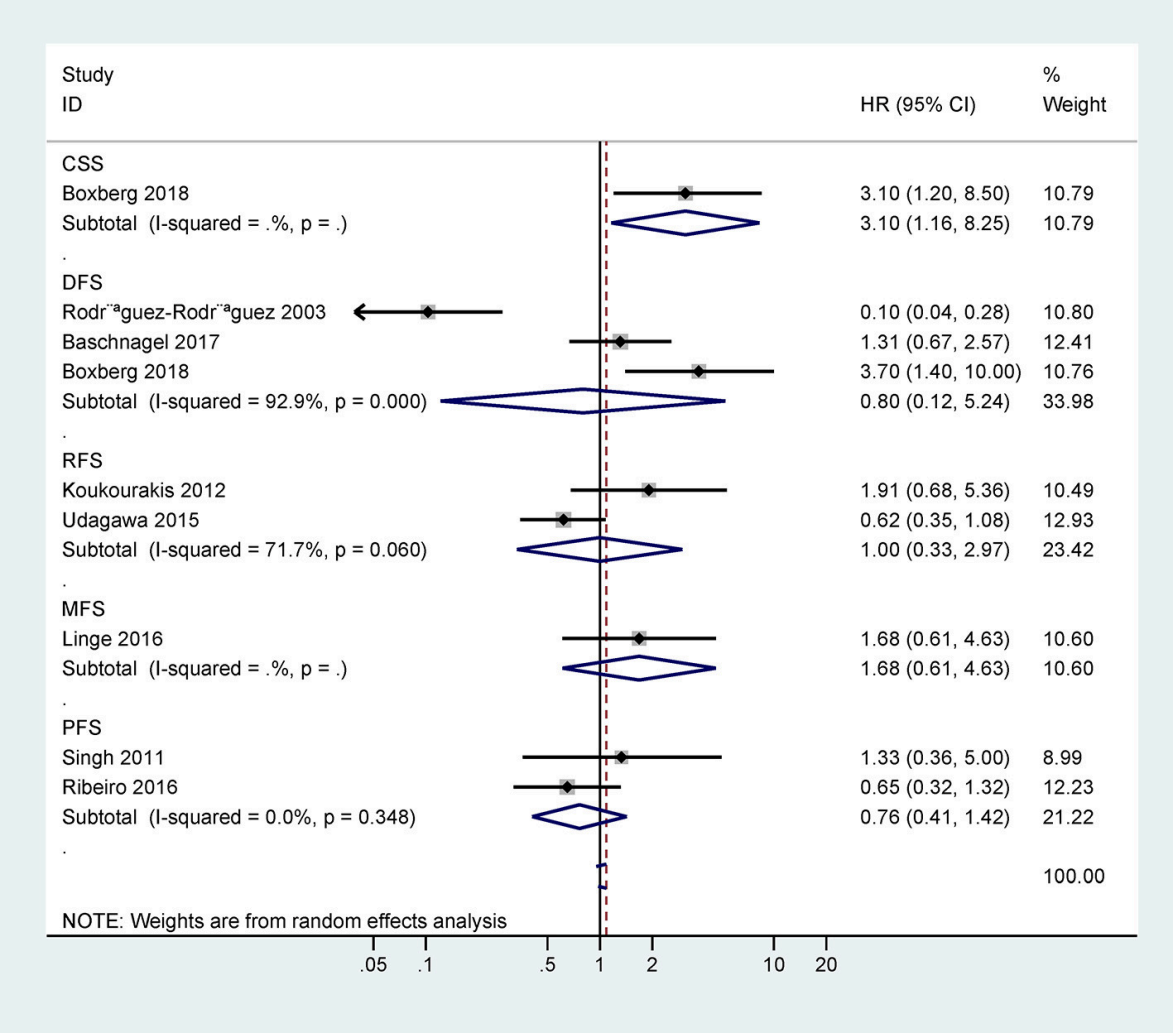
Forest plot for the association between CD44 expression and disease-free survival (DFS), progression-free survival (PFS), relapse/recurrence-free survival (RFS), metastasis-free survival (MFS), or cancer-specific survival (CSS).

Only one study with 63 cases reported that CD44 expression was correlated with poor 5-years CSS (HR = 3.1, 95% CI = 1.2–8.5) (Figure 3). However, there was no statistical significance between CD44 expression and DFS, RFS, MFS, or PFS (Figure 3).

### Association Between CD44v6 Expression and the Prognosis

**Figure 4:**
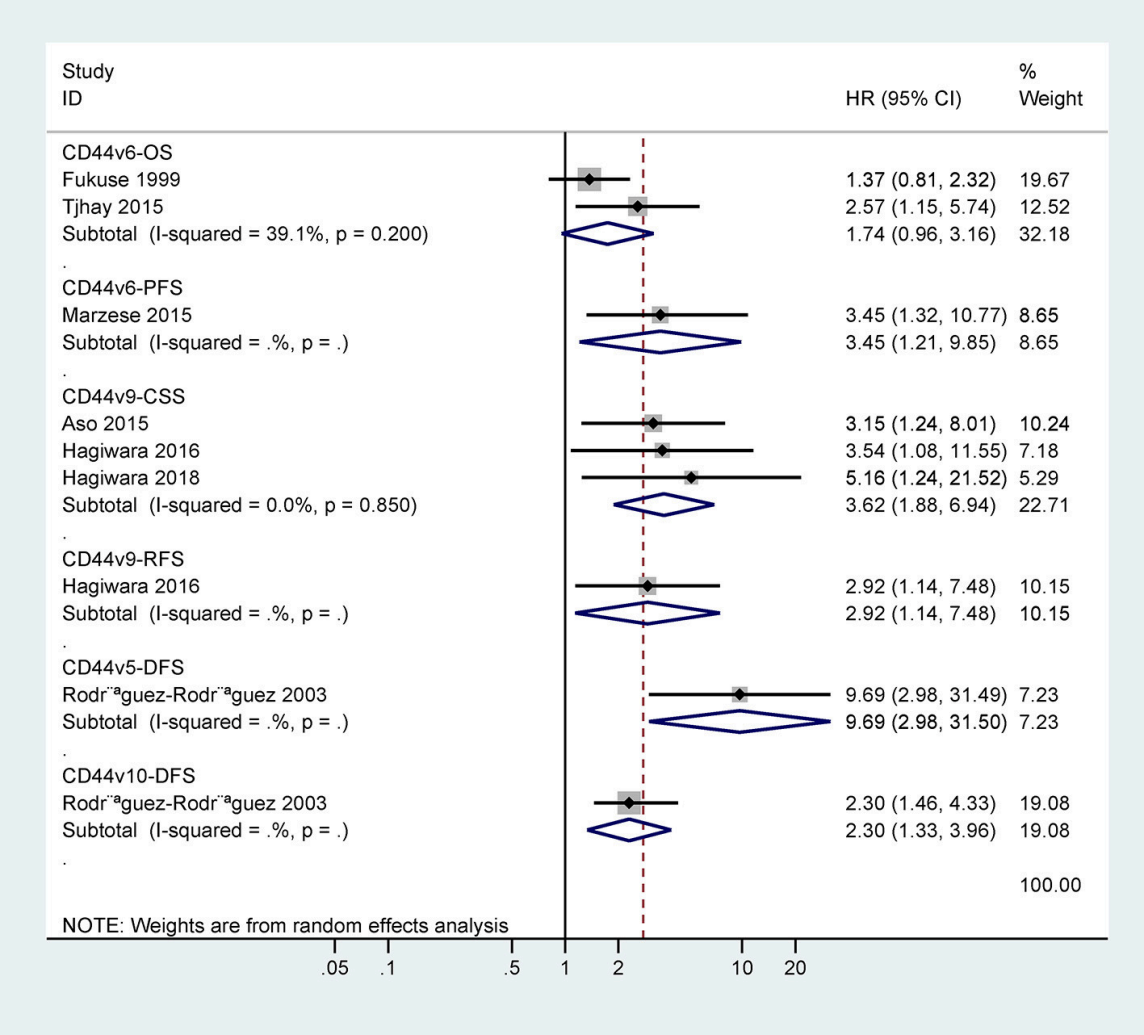
Forest plot for the association between CD44v6 or CD44v9 expression and the prognosis.

The data from two studies with 93 cases demonstrated no association between CD44v6 expression and 5-years OS (HR = 1.74, 95% CI = 0.96–3.16, *P* = 0.07) (Figure 4). One study involving 50 cases reported a significant association between CD44v6 expression and poor 5-years PFS (HR = 3.45, 95% CI = 1.32–10.77) (Figure 4).

### Association Between CD44v9 Expression and the Prognosis

Only one study with 83 cases reported that CD44v9 expression was linked to poor 5-years RFS (HR = 2.92, 95% CI = 1.14–7.48) (Figure 4). CD44v9 expression was significantly linked to worse 5-years CSS (HR = 3.62, 95% CI = 1.88–6.94, *P* < 0.001), including three studies with 262 cases receiving surgery and chemo/radiotherapy (Figure 4).

### TSA

**Figure 5:**
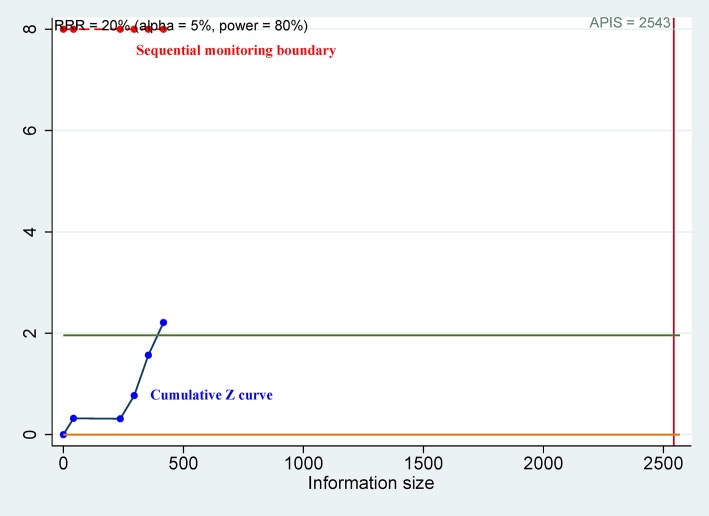
Trial sequential analysis (TSA) for overall survival (OS) of CD44.

**Figure 6:**
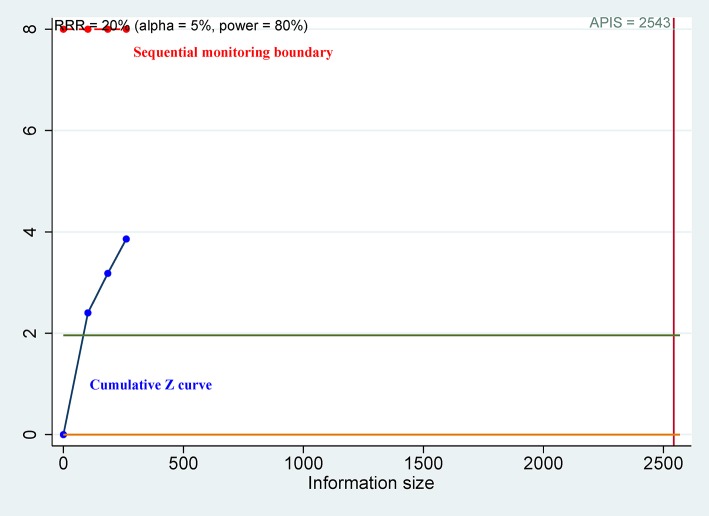
Trial sequential analysis (TSA) for cancer-specific survival (CSS) of CD44v9.

For OS of CD44, the cumulative Z curve did not reach the sequential monitoring boundary (Figure 5), a finding which indicated that more studies are needed to achieve the required information size. For CSS of CD44v9, the cumulative Z curve did not cross the trial sequential monitoring boundary (Figure 6), which demonstrated that additional studies were needed for stable conclusions.

## Discussion

Although advances in surgical techniques and treatment methods have been used for advanced cancer patients, the 5-years survival rate for these patients remains very disappointing. Drug resistance and frequent recurrence are the major obstacles to the treatment of advanced cancers (48, 49). According to the CSC theory, CSCs contribute to cancer progression, metastasis and chemotherapy/radiotherapy resistance (50, 51).

To date, CD44s remains the most commonly reported isoform in cancer, but the other CD44 variants (CD44v) are also correlated with neoplasia and metastasis in some cancers (52) CD44 is a frequently observed CSC marker in solid tumors, and CD44 was revealed to be a major Wnt target, involved in the Ras-Raf-Mek-Erk-Cyclin D1 pathway, phosphoinositide 3-kinase (PI3K)-Akt signaling, and the Wnt pathway, as well as stimulating EMT, which promotes tumor invasion, progression and metastasis (53–55). Some studies suggest that expression of total CD44 may be correlated with distant metastasis, tumor recurrence and a worse prognosis (19, 56, 57). CD44 or CD44v6 may also play a key role in drug resistance (39, 58, 59).

CD44v6 is one of the most common variants of CD44, and some meta-analyses have shown that the expression of CD44v6 is related to a poor prognosis in gastric and hepatocellular carcinomas (60, 61) but is not related to prognosis in ovarian cancer (20). CD44v9 may be correlated with lymph node/liver metastasis and tumor stage, and contribute to EMT-mediated invasion and migration (62, 63). Recent studies suggest that CD44v9 is associated with increased resistance to chemotherapy or radiation therapy and a poor prognosis in gastric cancer (64–66).

Despite numerous studies having investigated whether CD44, CD44v6, and CD44v9 may be potential prognostic indicators in many cancers, some findings are controversial. The impact of the expression of CD44, CD44v6, and CD44v9 on the prognosis of patients with advanced cancer has not been fully understood. Therefore, we conducted the first systematic meta-analysis to reveal the association of CD44 and its isoform CD44v6 and CD44v9 with the prognosis of advanced cancer patients by using multivariable survival data.

No statistical association was found between CD44 expression and DFS, RFS, MFS, or PFS. The expression of CD44 was not significantly associated with OS using multivariable analysis (35, 42, 43), but other studies showed a significant association between CD44 expression and shorter OS (45, 46). We integrated all eligible studies with a relatively large population, and found that CD44 expression was slightly linked to unfavorable OS of advanced cancer (HR = 2.03, *P* = 0.027). However, TSA suggested that this result was not reliable, and additional studies are needed.

To further investigate the impact of 5-years survival and adjuvant therapy, stratified analysis showed that CD44 expression was not correlated with OS at ≥5 years or in patients treated with adjuvant therapy. No relationship was reported between CD44v6 expression and OS in metastatic non-small cell lung carcinoma (33), but CD44v6 expression was correlated with worse OS in advanced epithelial ovarian cancer (39). Pooled results demonstrated no correlation between CD44v6 expression and OS in advanced cancer.

CD44v9 expression was closely associated with unfavorable CSS (37, 41, 47), and pooled data from three studies also showed a correlation in advanced cancer patients treated with surgery and chemo/radiotherapy (5-years CSS: HR = 3.62, *P* < 0.001). The above analyses suggested that CD44v9 may be a major variant of the CD44 family and play an important role in the prognosis of patients with advanced cancer. These results suggest that CD44v9 may provide more useful prognostic value for advanced cancer patient classification and survival benefit and could become a targeted selective treatment approach. However, we used TSA to check the reliability of these results, and TSA suggested that the available study population was insufficient to provide conclusive evidence. Additional studies are needed to further validate these conclusions in the future.

Although the analysis of the eligible studies revealed deficiencies in some items of the REMARK guidelines, in the present meta-analysis, the included studies based on multivariate survival analysis showed higher methodologic quality than studies based on univariate survival analysis. Several limitations should be acknowledged in this meta-analysis. First, the number of the included studies and sample sizes were relatively small, although all eligible studies were well-performed with data from multivariable survival analysis. Our conclusions should be interpreted with caution based on the TSA. Second, most studies were conducted in Japan and the USA and other study sources were lacking. Only one study was a prospective phase II trial and the other remaining studies were of retrospective design. Additional prospective clinical trials are necessary. The cut-off values of expression from the immunohistochemical studies may differ, and in the future, CD44s or CD44v expression should be defined as positive or negative based on a standard. Third, although CD44 expression was linked to worse CSS, CD44v6 expression was associated with worse PFS, CD44v9 was correlated with unfavorable RFS, and CD44v5 and CD44v10 were linked to poor DFS (34). These results were only reported by an individual study each. Finally, studies of different cancer types are strongly needed to further confirm the results regarding CD44s and CD44v1–v10 in advanced tumors.

In conclusion, the present study demonstrated that CD44 expression was linked to unfavorable OS, but there was no association between CD44 expression and OS at ≥5-years or OS in patients receiving adjuvant therapy. No association was found between CD44v6 expression and 5-years OS. CD44v9 expression was closely associated with worse 5-years CSS for patients with advanced cancer treated with surgery and chemo/radiotherapy, which suggested that CD44v9 may be a promising prognostic marker. TSA showed that these results cannot be considered conclusive. There is a need for predefined training and validation sets based on the REMARK guidelines, and additional prospective clinical trials in advanced cancer are necessary to further determine whether CD44s and CD44v (v1–v10) may help stratify different cancer patients who could benefit from chemo/radiotherapy.

## Ethics Statement

The present study was not primary research involving human samples in the public databases.

## Author Contributions

SH, TH, and FH contributed to the conception and design of this research. SH, XWu, XWa, WL, SL, WY, QS, and HL contributed to the drafting of the article and final approval of the submitted version. SH, TH, XWu, XWa, WL, SL, WY, QS, HL, and FH contributed to data analyses and the interpretation and completion of the figures and tables. All authors read and approved the final manuscript.

### Conflict of Interest Statement

The authors declare that the research was conducted in the absence of any commercial or financial relationships that could be construed as a potential conflict of interest.

## Supplementary Material

Table S1 REMARK guidelines.

Table S2 Detailed characteristics of the eligible studies in the meta-analysis.

## References

[1] Torre LA Bray F Siegel RL Ferlay J Lortet-Tieulent J Jemal A. Global cancer statistics, 2012. CA Cancer J Clin. (2015) 65:87–108. 10.3322/caac.21262 25651787

[2] Urruticoechea A Alemany R Balart J Villanueva A Vinals F Capella G. Recent advances in cancer therapy: an overview. Curr Pharm Des. (2010) 16:3–10. 10.2174/138161210789941847 20214614

[3] Solomon BJ Mok T Kim DW Wu YL Nakagawa K Mekhail T. First-line crizotinib versus chemotherapy in ALK-positive lung cancer. N Engl J Med. (2014) 371:2167–77. 10.1056/NEJMoa1408440 25470694

[4] Farkona S Diamandis EP Blasutig IM. Cancer immunotherapy: the beginning of the end of cancer? BMC Med. (2016) 14:73. 10.1186/s12916-016-0623-5 27151159 PMC4858828

[5] Martinelli E Morgillo F Troiani T Ciardiello F. Cancer resistance to therapies against the EGFR-RAS-RAF pathway: the role of MEK. Cancer Treat Rev. (2017) 53:61–9. 10.1016/j.ctrv.2016.12.001 28073102

[6] Pasquali S Hadjinicolaou AV Chiarion Sileni V Rossi CR Mocellin S. Systemic treatments for metastatic cutaneous melanoma. Cochrane Database Syst Rev. (2018) 2:CD011123. 10.1002/14651858.CD011123.pub2 29405038 PMC6491081

[7] Siegel RL Miller KD Jemal A Cancer statistics, 2018. CA Cancer J Clin. (2018) 68:7–30. 10.3322/caac.21442 29313949

[8] Reya T Morrison SJ Clarke MF Weissman IL. Stem cells, cancer, and cancer stem cells. Nature (2001) 414:105–11. 10.1038/35102167 11689955

[9] Liau BB Sievers C Donohue LK Gillespie SM Flavahan WA Miller TE. Adaptive chromatin remodeling drives glioblastoma stem cell plasticity and drug tolerance. Cell Stem Cell (2017) 20:233–46 e237. 10.1016/j.stem.2016.11.003 27989769 PMC5291795

[10] Yan Y Zuo X Wei D. Concise review: emerging role of CD44 in cancer stem cells: a promising biomarker and therapeutic target. Stem Cells Transl Med. (2015) 4:1033–43. 10.5966/sctm.2015-0048 26136504 PMC4542874

[11] Han S Yang W Zong S Li H Liu S Li W. Clinicopathological, prognostic and predictive value of CD166 expression in colorectal cancer: a meta-analysis. Oncotarget (2017a) 8:64373–84. 10.18632/oncotarget.17442 28969077 PMC5610009

[12] Han S Zong S Shi Q Li H Liu S Yang W. Is Ep-CAM expression a diagnostic and prognostic biomarker for colorectal cancer? A systematic meta-analysis. EBioMed. (2017b) 20:61–9. 10.1016/j.ebiom.2017.05.025 28558958 PMC5478257

[13] Naor D Sionov RV Ish-Shalom D. CD44: structure, function, and association with the malignant process. Adv Cancer Res. (1997) 71:241–319. 10.1016/S0065-230X(08)60101-3 9111868

[14] Brown RL Reinke LM Damerow MS Perez D Chodosh LA Yang J. CD44 splice isoform switching in human and mouse epithelium is essential for epithelial-mesenchymal transition and breast cancer progression. J Clin Invest. (2011) 121:1064–74. 10.1172/JCI44540 21393860 PMC3049398

[15] Negi LM Talegaonkar S Jaggi M Ahmad FJ Iqbal Z Khar RK. Role of CD44 in tumour progression and strategies for targeting. J Drug Target. (2012) 20:561–73. 10.3109/1061186X.2012.702767 22758394

[16] Sacks JD Barbolina MV. Expression and function of CD44 in epithelial ovarian carcinoma. Biomolecules (2015) 5:3051–66. 10.3390/biom5043051 26569327 PMC4693269

[17] Zhao S Chen C Chang K Karnad A Jagirdar J Kumar AP. CD44 Expression level and isoform contributes to pancreatic cancer cell plasticity, invasiveness, and response to therapy. Clin Cancer Res. (2016b) 22:5592–604. 10.1158/1078-0432.CCR-15-3115 27267855 PMC5143222

[18] Nagano O Okazaki S Saya H. Redox regulation in stem-like cancer cells by CD44 variant isoforms. Oncogene (2013) 32:5191–8. 10.1038/onc.2012.638 23334333

[19] Fang M Wu J Lai X Ai H Tao Y Zhu B. CD44 and CD44v6 are correlated with gastric cancer progression and poor patient prognosis: evidence from 42 studies. Cell Physiol Biochem. (2016) 40:567–78. 10.1159/000452570 27889771

[20] Zhao L Gu C Huang K Zhang Z Ye M Fan W. The prognostic value and clinicopathological significance of CD44 expression in ovarian cancer: a meta-analysis. Arch Gynecol Obstet. (2016a) 294:1019–29. 10.1007/s00404-016-4137-3 27300001

[21] Liberati A Altman DG Tetzlaff J Mulrow C Gotzsche PC Ioannidis JP. The PRISMA statement for reporting systematic reviews and meta-analyses of studies that evaluate health care interventions: explanation and elaboration. PLoS Med. (2009) 6:e1000100. 10.1371/journal.pmed.1000100 19621070 PMC2707010

[22] Tierney JF Stewart LA Ghersi D Burdett S Sydes MR. Practical methods for incorporating summary time-to-event data into meta-analysis. Trials (2007) 8:16. 10.1186/1745-6215-8-16 17555582 PMC1920534

[23] Altman DG Bland JM How to obtain the confidence interval from a P value. BMJ (2011) 343:d2090 10.1136/bmj.d2090 21824904

[24] McShane LM Altman DG Sauerbrei W Taube SE Gion M Clark GM. Reporting recommendations for tumor marker prognostic studies (REMARK). J Natl Cancer Inst. (2005) 97:1180–4. 10.1093/jnci/dji237 16106022

[25] Zintzaras E Ioannidis JP. HEGESMA: genome search meta-analysis and heterogeneity testing. Bioinformatics (2005) 21:3672–3. 10.1093/bioinformatics/bti536 15955784

[26] DerSimonian R Kacker R. Random-effects model for meta-analysis of clinical trials: an update. Contemp Clin Trials (2007) 28:105–14. 10.1016/j.cct.2006.04.004 16807131

[27] Evangelou E Ioannidis JP. Meta-analysis methods for genome-wide association studies and beyond. Nat Rev Genet. (2013) 14:379–89. 10.1038/nrg3472 23657481

[28] Begg CB Mazumdar M. Operating characteristics of a rank correlation test for publication bias. Biometrics (1994) 50:1088–101. 10.2307/2533446 7786990

[29] Egger M Davey Smith G Schneider M Minder C. Bias in meta-analysis detected by a simple, graphical test. BMJ (1997) 315:629–34. 10.1136/bmj.315.7109.629 9310563 PMC2127453

[30] Thorlund K Devereaux PJ Wetterslev J Guyatt G Ioannidis JP Thabane L. Can trial sequential monitoring boundaries reduce spurious inferences from meta-analyses? Int J Epidemiol. (2009) 38:276–86. 10.1093/ije/dyn179 18824467

[31] Miladinovic B Mhaskar R Hozo I Kumar A Mahony H Djulbegovic B. Optimal information size in trial sequential analysis of time-to-event outcomes reveals potentially inconclusive results because of the risk of random error. J Clin Epidemiol. (2013) 66:654–9. 10.1016/j.jclinepi.2012.11.007 23403248

[32] Brok J Thorlund K Gluud C Wetterslev J. Trial sequential analysis reveals insufficient information size and potentially false positive results in many meta-analyses. J Clin Epidemiol. (2008) 61:763–9. 10.1016/j.jclinepi.2007.10.007 18411040

[33] Fukuse T Hirata T Naiki H Hitomi S Wada H. Expression of proliferating cell nuclear antigen and CD44 variant isoforms in the primary and metastatic sites of nonsmall cell lung carcinoma with intrapulmonary metastases. Cancer (1999) 86:1174–81. 10506701 10.1002/(sici)1097-0142(19991001)86:7<1174::aid-cncr11>3.0.co;2-8

[34] Rodriguez-Rodriguez L Sancho-Torres I Mesonero C Gibbon DG Shih WJ Zotalis G. The CD44 receptor is a molecular predictor of survival in ovarian cancer. Med Oncol. (2003) 20:255–63. 10.1385/MO:20:3:255 14514975

[35] Singh M Darcy KM Brady WE Clubwala R Weber Z Rittenbach JV. Cadherins, catenins and cell cycle regulators: impact on survival in a Gynecologic Oncology Group phase II endometrial cancer trial. Gynecol Oncol. (2011) 123:320–8. 10.1016/j.ygyno.2011.07.005 21813170 PMC3518446

[36] Koukourakis MI Giatromanolaki A Tsakmaki V Danielidis V Sivridis E. Cancer stem cell phenotype relates to radio-chemotherapy outcome in locally advanced squamous cell head-neck cancer. Br J Cancer (2012) 106:846–53. 10.1038/bjc.2012.33 22333601 PMC3305970

[37] Aso T Matsuo M Kiyohara H Taguchi K Rikimaru F Shimokawa M. Induction of CD44 variant 9-expressing cancer stem cells might attenuate the efficacy of chemoradioselection and Worsens the prognosis of patients with advanced head and neck cancer. PLoS ONE (2015) 10:e0116596. 10.1371/journal.pone.0116596 25751671 PMC4353624

[38] Marzese DM Liu M Huynh JL Hirose H Donovan NC Huynh KT. Brain metastasis is predetermined in early stages of cutaneous melanoma by CD44v6 expression through epigenetic regulation of the spliceosome. Pigment Cell Melanoma Res. (2015) 28:82–93. 10.1111/pcmr.12307 25169209 PMC4309554

[39] Tjhay F Motohara T Tayama S Narantuya D Fujimoto K Guo J. CD44 variant 6 is correlated with peritoneal dissemination and poor prognosis in patients with advanced epithelial ovarian cancer. Cancer Sci. (2015) 106:1421–8. 10.1111/cas.12765 26250934 PMC4638001

[40] Udagawa H Ishii G Morise M Umemura S Matsumoto S Yoh K. Comparison of the expression levels of molecular markers among the peripheral area and central area of primary tumor and metastatic lymph node tumor in patients with squamous cell carcinoma of the lung. J Cancer Res Clin Oncol. (2015) 141:1417–25. 10.1007/s00432-015-1912-7 25573625 PMC11823983

[41] Hagiwara M Kikuchi E Kosaka T Mikami S Saya H Oya M. Variant isoforms of CD44 expression in upper tract urothelial cancer as a predictive marker for recurrence and mortality. Urol Oncol. (2016) 34:337 e319–26. 10.1016/j.urolonc.2016.03.015 27133224

[42] Linge A Lock S Gudziol V Nowak A Lohaus F von Neubeck C. Low cancer stem cell marker expression and low hypoxia identify good prognosis subgroups in HPV(-) HNSCC after postoperative radiochemotherapy: a multicenter study of the DKTK-ROG. Clin Cancer Res. (2016) 22:2639–49. 10.1158/1078-0432.CCR-15-1990 26755529

[43] Ribeiro KB da Silva Zanetti J Ribeiro-Silva A Rapatoni L de Oliveira HF da Cunha Tirapelli DP. KRAS mutation associated with CD44/CD166 immunoexpression as predictors of worse outcome in metastatic colon cancer. Cancer Biomark. (2016) 16:513–21. 10.3233/CBM-160592 27062566 PMC13016524

[44] Baschnagel AM Tonlaar N Eskandari M Kumar T Williams L Hanna A. Combined CD44, c-MET, and EGFR expression in p16-positive and p16-negative head and neck squamous cell carcinomas. J Oral Pathol Med. (2017) 46:208–13. 10.1111/jop.12478 27442811

[45] Sun H Liu T Zhu D Dong X Liu F Liang X. HnRNPM and CD44s expression affects tumor aggressiveness and predicts poor prognosis in breast cancer with axillary lymph node metastases. Genes Chromosomes Cancer (2017) 56:598–607. 10.1002/gcc.22463 28393427

[46] Boxberg M Gotz C Haidari S Dorfner C Jesinghaus M Drecoll E Immunohistochemical expression of CD44 in oral squamous cell carcinoma in relation to histomorphologic parameters and clinicopathological factors. Histopathology (2018) 73:559–72. 10.1111/his.13496 29468726

[47] Hagiwara M Kikuchi E Tanaka N Kosaka T Mikami S Saya H. Variant isoforms of CD44 involves acquisition of chemoresistance to cisplatin and has potential as a novel indicator for identifying a cisplatin-resistant population in urothelial cancer. BMC Cancer (2018) 18:113. 10.1186/s12885-018-3988-3 29385995 PMC5793458

[48] Mentha G Terraz S Andres A Toso C Rubbia-Brandt L Majno P. Operative management of colorectal liver metastases. Semin Liver Dis. (2013) 33:262–72. 10.1055/s-0033-1351785 23943106

[49] Frezza AM Stacchiotti S Gronchi A. Systemic treatment in advanced soft tissue sarcoma: what is standard, what is new. BMC Med. (2017) 15:109. 10.1186/s12916-017-0872-y 28571564 PMC5455204

[50] Morrison R Schleicher SM Sun Y Niermann KJ Kim S Spratt DE. Targeting the mechanisms of resistance to chemotherapy and radiotherapy with the cancer stem cell hypothesis. J Oncol. (2011) 2011:941876. 10.1155/2011/941876 20981352 PMC2958340

[51] Nguyen LV Vanner R Dirks P Eaves CJ. Cancer stem cells: an evolving concept. Nat Rev Cancer (2012) 12:133–43. 10.1038/nrc3184 22237392

[52] Rajarajan A Stokes A Bloor BK Ceder R Desai H Grafstrom RC. CD44 expression in oro-pharyngeal carcinoma tissues and cell lines. PLoS ONE (2012) 7:e28776. 10.1371/journal.pone.0028776 22242150 PMC3252301

[53] Zoller M. CD44: can a cancer-initiating cell profit from an abundantly expressed molecule? Nat Rev Cancer (2011) 11:254–67. 10.1038/nrc3023 21390059

[54] Xu H Tian Y Yuan X Wu H Liu Q Pestell RG. The role of CD44 in epithelial-mesenchymal transition and cancer development. Onco Targets Ther. (2015) 8:3783–92. 10.2147/OTT.S95470 26719706 PMC4689260

[55] Hasebe T Fujimoto K Kajita M Ishizuya-Oka A. Essential roles of thyroid hormone-regulated hyaluronan/CD44 signaling in adult stem cell development during *Xenopus laevis* intestinal remodeling. Stem Cells (2017) 35:2175–83. 10.1002/stem.2671 28758360

[56] Chen Y Fu Z Xu S Xu Y Xu P. The prognostic value of CD44 expression in gastric cancer: a meta-analysis. Biomed Pharmacother. (2014) 68:693–7. 10.1016/j.biopha.2014.08.001 25194445

[57] Li X Ma X Chen L Gu L Zhang Y Zhang F. Prognostic value of CD44 expression in renal cell carcinoma: a systematic review and meta-analysis. Sci Rep. (2015) 5:13157. 10.1038/srep13157 26287771 PMC4541415

[58] Palyi-Krekk Z Barok M Isola J Tammi M Szollosi J Nagy P. Hyaluronan-induced masking of ErbB2 and CD44-enhanced trastuzumab internalisation in trastuzumab resistant breast cancer. Eur J Cancer (2007) 43:2423–33. 10.1016/j.ejca.2007.08.018 17911008

[59] Zhang X He F Xiang K Zhang J Xu M Long P. CD44-Targeted facile enzymatic activatable chitosan nanoparticles for efficient antitumor therapy and reversal of multidrug resistance. Biomacromolecules (2018) 19:883–95. 10.1021/acs.biomac.7b01676 29401378

[60] Fu Y Geng Y Yang N Zhu N Wang CZ Su XC. CD44v6 expression is associated with a poor prognosis in Chinese hepatocellular carcinoma patients: a meta-analysis. Clin Res Hepatol Gastroenterol. (2015) 39:736–9. 10.1016/j.clinre.2015.03.001 25887688

[61] Lu L Huang F Zhao Z Li C Liu T Li W. CD44v6: a metastasis-associated biomarker in patients with gastric cancer?: a comprehensive meta-analysis with heterogeneity analysis. Medicine (2016) 95:e5603. 10.1097/MD.0000000000005603 27977599 PMC5268045

[62] Li Z Chen K Jiang P Zhang X Li X Li Z. CD44v/CD44s expression patterns are associated with the survival of pancreatic carcinoma patients. Diagn Pathol. (2014) 9:79. 10.1186/1746-1596-9-79 24708709 PMC4108087

[63] Kobayashi K Matsumoto H Matsuyama H Fujii N Inoue R Yamamoto Y. Clinical significance of CD44 variant 9 expression as a prognostic indicator in bladder cancer. Oncol Rep. (2016) 36:2852–60. 10.3892/or.2016.5061 27599396

[64] Kodama H Murata S Ishida M Yamamoto H Yamaguchi T Kaida S. Prognostic impact of CD44-positive cancer stem-like cells at the invasive front of gastric cancer. Br J Cancer (2017) 116:186–94. 10.1038/bjc.2016.401 27931044 PMC5243989

[65] Yamakawa Y Kusuhara M Terashima M Kinugasa Y Sugino T Abe M. CD44 variant 9 expression as a predictor for gastric cancer recurrence: immunohistochemical and metabolomic analysis of surgically resected tissues. Biomed Res. (2017) 38:41–52. 10.2220/biomedres.38.41 28239031

[66] Zavros Y. Initiation and maintenance of gastric cancer: a focus on CD44 variant isoforms and cancer stem cells. Cell Mol Gastroenterol Hepatol. (2017) 4:55–63. 10.1016/j.jcmgh.2017.03.003 28560289 PMC5439237

