# Supplementary material for: Prognostic Value of CD44 and Its Isoforms in Advanced Cancer: A Systematic Meta-Analysis With Trial Sequential Analysis
Source: Front Oncol. 2019 Feb 6;9:39. doi: 10.3389/fonc.2019.00039 (PMC6372530; doi:10.3389/fonc.2019.00039)
Supplement: Table S1 — REMARK guidelines. [file Table_1.DOCX]

**Table S1 REMARK guidelines**

| **Author** | **1** | **2** | **3** | **4** | **5** | **6** | **7** | **8** | **9** | **10** | **11** | **12** | **13** | **14** | **15** | **16** | **17** | **18** | **19** | **20** | **Total** |
| --- | --- | --- | --- | --- | --- | --- | --- | --- | --- | --- | --- | --- | --- | --- | --- | --- | --- | --- | --- | --- | --- |
| Rodríguez 2003 | 2 | 1 | 0 | 1 | 1 | 1 | 0 | 2 | 0 | 1 | 0 | 0 | 1 | 0 | 2 | 2 | 1 | 0 | 1 | 1 | 17 |
| Singh 2011 | 1 | 2 | 2 | 1 | 1 | 1 | 1 | 2 | 0 | 1 | 1 | 1 | 1 | 1 | 2 | 2 | 1 | 0 | 2 | 0 | 23 |
| Koukourakis 2012 | 1 | 1 | 1 | 1 | 1 | 1 | 0 | 1 | 0 | 1 | 0 | 0 | 0 | 2 | 2 | 1 | 1 | 0 | 1 | 0 | 15 |
| Udagawa 2015 | 0 | 2 | 1 | 1 | 1 | 1 | 2 | 0 | 0 | 1 | 0 | 0 | 2 | 0 | 2 | 0 | 2 | 0 | 1 | 0 | 16 |
| Linge 2016 | 1 | 2 | 2 | 1 | 1 | 1 | 1 | 1 | 0 | 1 | 2 | 0 | 0 | 0 | 2 | 2 | 2 | 0 | 1 | 1 | 21 |
| Ribeiro 2016 | 2 | 1 | 1 | 1 | 1 | 1 | 2 | 0 | 0 | 1 | 1 | 0 | 1 | 2 | 1 | 2 | 2 | 0 | 1 | 1 | 21 |
| Baschnagel 2017 | 2 | 1 | 1 | 1 | 1 | 1 | 2 | 2 | 0 | 1 | 0 | 1 | 1 | 1 | 2 | 2 | 2 | 0 | 1 | 2 | 24 |
| Sun 2017 | 2 | 1 | 1 | 1 | 1 | 1 | 2 | 0 | 0 | 0 | 0 | 0 | 0 | 0 | 2 | 1 | 0 | 0 | 1 | 1 | 14 |
| Boxberg 2018 | 2 | 2 | 1 | 1 | 1 | 1 | 0 | 0 | 0 | 1 | 0 | 0 | 1 | 0 | 1 | 2 | 2 | 0 | 2 | 1 | 18 |
| Aso 2015 | 1 | 2 | 2 | 1 | 1 | 1 | 0 | 1 | 0 | 1 | 0 | 0 | 1 | 0 | 2 | 2 | 2 | 0 | 1 | 1 | 19 |
| Hagiwara 2016 | 2 | 2 | 2 | 1 | 1 | 1 | 1 | 2 | 0 | 1 | 0 | 0 | 1 | 0 | 2 | 2 | 0 | 0 | 2 | 1 | 21 |
| Hagiwara 2018 | 2 | 2 | 2 | 1 | 1 | 1 | 1 | 2 | 0 | 1 | 0 | 0 | 1 | 2 | 1 | 2 | 0 | 0 | 1 | 1 | 21 |
| Fukuse 1999 | 0 | 2 | 1 | 1 | 1 | 1 | 0 | 0 | 0 | 1 | 0 | 1 | 0 | 2 | 0 | 2 | 2 | 0 | 1 | 1 | 16 |
| Marzese 2015 | 2 | 1 | 0 | 1 | 1 | 0 | 0 | 2 | 0 | 1 | 2 | 1 | 1 | 0 | 2 | 2 | 1 | 0 | 1 | 1 | 19 |
| Tjhay 2015 | 2 | 2 | 1 | 1 | 1 | 1 | 0 | 0 | 0 | 1 | 0 | 0 | 1 | 2 | 2 | 2 | 1 | 0 | 1 | 1 | 19 |
