# Supplementary material for: Prognostic Value of CD44 and Its Isoforms in Advanced Cancer: A Systematic Meta-Analysis With Trial Sequential Analysis
Source: Front Oncol. 2019 Feb 6;9:39. doi: 10.3389/fonc.2019.00039 (PMC6372530; doi:10.3389/fonc.2019.00039)
Supplement: Table S2 — Detailed characteristics of the eligible studies in the meta-analysis. [file Table_2.DOCX]

**Table S2 Detailed characteristics of the eligible studies in the meta-analysis**

|  | First author | Country | Median follow-up time | Age | Method | Cancer type | Study design | Specimen  type | Cases | Therapy | Staining  patterns | Cut off (positivity) | Survival rate | Adjusted variables | Outcomes | REMARK scores |
| --- | --- | --- | --- | --- | --- | --- | --- | --- | --- | --- | --- | --- | --- | --- | --- | --- |
| CD44 |  |  |  |  |  |  |  |  |  |  |  |  |  |  |  |  |
|  | Rodríguez 2003 | USA | 1-92 months | NA | IHC | Metastatic ovarian cancer | NA, single-center | Paraffin-embedded tumor specimens or fresh frozen tumor tissue | 87 | NA | R&D Systems, MN; dilution 1:1000 | Membrane/cytoplasm 0% | < 5 years | Stage, histology, and age | DFS | 17 |
|  | Singh 2011 | USA | NA | NA | IHC, blind | Metastatic or recurrent endometrial carcinoma | Prospective phase II trial, Gynecologic Oncology Group | Paraffin-embedded  tumor specimens | 42 | Tamoxifen combined with intermittent medroxyprogesterone acetate | Dako anti-CD44 clone DF1485 | Cytoplasm median-high | NA | Patient age at study enrollment  and stratification for performance status and tumor grade | OS, PFS | 23 |
|  | Koukourakis 2012 | Greece | 24 months (4-80) | 68 | IHC, blind | Advanced squamous cell head-neck cancer | NA, multicentre | Paraffin-embedded tumor specimens | 74 | Surgery and radio-chemotherapy | Ab6124, Abcam, Cambridge, UK; dilution 1:100 | Membrane/cytoplasm 40% | 5 years | T-stage, N-stage, and grade | RFS | 15 |
|  | Udagawa 2015 | Japan | 8.6 years (0.3-16.3) | 66 | IHC | Lung squamous cell carcinoma with lymph node metastasis | NA, single-center | Paraffin-embedded  tumor specimens | 113 | Surgery | Clone DF1485, Novocastra Laboratories; dilution 1:150 | NA median | 5 years | Lymph node factor | RFS | 16 |
|  | Linge 2016 | Germany | 47.3 months | NA | IHC, blind | Advanced head and neck squamous cell carcinoma | Retrospective, multicenter | Paraffin-embedded tumor specimens | 195 | Surgery and cisplatin-based radiochemotherapy | clone DF1485; Dako; dilution 1:500 | NA 1 score | 5 years | HPV16 DNA, extracapsular extension status, and tumor localization | OS, MFS | 21 |
|  | Ribeiro 2016 | Brazil | 42 months (34-60) | NA | IHC | Metastatic colon cancer | Retrospective, single-center | Paraffin-embedded tumor specimens | 58 | Chemotherapy | Clone DF1485, DakoCytomation | Membrane and/or cytoplasm 3-6 scores | NA | ECOG, synchronous metastases, lymph node involvement, category tumor extension, and KRAS | OS, PFS | 21 |
|  | Baschnagel 2017 | USA | 35 months (1-93) | 61 | IHC, blind | Advanced head and neck squamous cell carcinoma | Retrospective, NA | Paraffin-embedded tumor specimens | 105 | Chemoradiotherapy | Clone EPR1013Y, Abcam, Cambridge, MA, USA, dilution 1:50 | 50% of tumor cells | 5 years | T-Stage, smoking, and concurrent chemotherapy | DFS | 24 |
|  | Sun 2017 | China | 17-148 months | NA | IHC | Breast cancer with axillary lymph node metastasis | NA, single-center | Paraffin-embedded tumor specimens or fresh frozen tumor tissue | 59 | Surgery | Peking Zhongshan Biotechnology Limited Company, China | NA > 3 scores | > 5 years | Patients’age, grade, ER, PR, and Her2 | OS | 14 |
|  | Boxberg 2018 | Germany | 3-66 months | NA | IHC | Advanced oral squamous cell carcinoma | Retrospective, single-center | Paraffin-embedded tumor specimens or fresh frozen tumor tissue | 63 | Surgery and adjuvant radiotherapy | Cell Signaling, clone 156-3C11; dilution 1:100 | Membrane 9-12 scores | 5 years | Age, gender, grading, pT- and pN-stage | CSS, OS, DFS | 18 |
| CD44v9 |  |  |  |  |  |  |  |  |  |  |  |  |  |  |  |  |
|  | Aso 2015 | Japan | Mean 51.7 months (2-151) | 60.8 | IHC | Advanced head and neck squamous cell carcinoma | NA, National Kyushu Cancer Center | Paraffin-embedded tumor specimens | 102 | Surgery and chemoradiotherapy | RV3; dilution 1:12500 | 2-5 scores | 5 years | T stage, N stage, and treatment effect | CSS | 19 |
|  | Hagiwara 2016 | Japan | Mean 57 months | NA | IHC, blind | Advanced upper tract urothelial cancer | Retrospective, single-center | Paraffin-embedded tumor specimens | 83 | Surgery and adjuvant cisplatin-based chemotherapy | CosmoBio,Tokyo,Japan; dilution 1:5000 | Cell membrane 5% | 5 years | Tumor location and tumor grade | CSS, RFS | 21 |
|  | Hagiwara 2018 | Japan | Mean 45 months | 68 | IHC, blind | Metastatic and/or recurrent urothelial cancer | Retrospective, single-center | Paraffin-embedded tumor specimens | 77 | Surgery and cisplatin-based chemotherapy | Cosmo Bio, Tokyo, Japan; dilution 1:5000 | Cell membrane 5% | 5 years | Age, gender, tumor location, tumor grade, pathological T stage, lymphovascular invasion, and lymph node metastasis | CSS | 21 |
| CD44v6 |  |  |  |  |  |  |  |  |  |  |  |  |  |  |  |  |
|  | Fukuse 1999 | Japan | Mean 64 months | 64.3 | IHC, blind | Metastatic nonsmall cell lung carcinoma | Retrospective; NA | Paraffin-embedded tumor specimens | 34 | Surgery and adjuvant therapies | VFF-18; Bender Co; dilution 1:100 | Cell membrane 20% | 5 years | Age, gender, pathologic T (pT) classification, and pathologic N (pN) classification | OS | 16 |
|  | Marzese 2015 | USA | NA | 62.3 | IHC | Metastatic melanoma | NA, single-center | Paraffin-embedded tumor specimens | 50 | Surgery | H-CAM; Santa Cruz Biotechnology, Santa Cruz, CA | 27 | 5 years | Breslow thickness, ulceration, primary tumor location, age, and gender | PFS | 19 |
|  | Tjhay 2015 | Japan | NA | 57 | IHC | Advanced epithelial ovarian cancer | Retrospective; single-center | Paraffin-embedded tumor specimens | 59 | Surgery and chemotherapy | 2F10; R&D Systems, Minneapolis, MN, USA | 10% | 5 years | Age, CA125, tumor size, chemotherapy, and surgery status | OS | 19 |

IHC: immunohistochemistry; NA: not applicable; T-stage: pathologic tumor (pT) classification; N-stage: pathologic lymph node (pN) classification; HPV16 DNA: human papillomavirus type 16 deoxyribonucleic acid; ECOG: Eastern Cooperative Oncology Group; KRAS: Kirsten rat sarcoma viral oncogene; ER: estrogen receptor; HER2: human epidermal growth factor receptor-2; PR: progesterone receptor; CA125: carbohydrate antigen 125; REMARK: Reporting Recommendations for Tumor Marker Prognostic Studies; OS: overall survival; DFS: disease-free survival; PFS: progression-free survival; CSS: cancer-specific survival; RFS: recurrence-free survival (RFS); MFS: metastasis-free survival.
